# Pain assessment: the relationship between pain thresholds and pain severity in osteoarthritis

**DOI:** 10.1186/1745-6215-12-S1-A79

**Published:** 2011-12-13

**Authors:** Vikki Wylde, Shea Palmer, Ian D Learmonth, Paul Dieppe

**Affiliations:** 1 University of Bristol, Bristol, BS10 5NB, UK; 2 University of the West of England, Bristol, BS16 1QY, UK; 3 Peninsula Medical School, Universities of Exeter and Plymouth, PL6 8BU, UK

## Objectives

When pain is assessed in research, it is most commonly assessed using patient-reported outcome measures (PROMS). These measures can provide information about pain severity, distress and qualities, but they are unable to provide information about the underlying mechanisms contributing to pain perception. Quantitative Sensory Testing (QST) comprises non-invasive tests which can identify abnormalities in pain processing on a localised and widespread level. The aim of this study was to explore the relationship between the measurement of pain thresholds by two different QST methods and the measurement of pain severity by a validated PROM.

## Methods

Patients on the waiting list for a total knee replacement because of osteoarthritis were approached about this study, and 107 patients attended a QST session**.** A digital algometer (Somedic) with a 1cm probe was used to assess pressure pain thresholds (in kPa) three times at each site. A MSA Thermotest (Somedic) was used to measure hot pain thresholds (in Celsius) four times at each site. Pain thresholds were tested at the painful knee and the pain-free forearm. Self-reported pain severity was assessed using the WOMAC questionnaire. Spearman-Rank Correlation Coefficients (CC) were calculated to determine the strength of correlation between pain thresholds and pain severity.

## Results

Pain severity in the knee significantly correlated with pressure pain thresholds at the knee (CC=0.275, p=0.005) and the forearm (CC=0.208, p=0.03). No significant correlation was found between pain severity in the knee and heat pain thresholds at the knee (CC=0.118, p=0.236) or the forearm (CC=0.134; p=0.173).

## Conclusions

This study found a small but significant positive correlation between self-reported knee pain severity and pressure pain thresholds, but not heat pain thresholds. This suggests that as pain worsens, patients become more sensitive to pressure pain at the osteoarthritic knee and the pain-free forearm. Increased pain sensitivity at a pain-free distant body site, such as the forearm, suggests involvement of the central nervous system and widespread pain sensitisation. The addition of Algometry as an outcome measure in clinical trials and pain studies could improve understanding of the mechanisms contributing to self-reported pain severity, by identifying the presence and extent of pain sensitisation. We are currently using QST in a large randomised controlled trial of pain control in arthroplasty (the APEX study), to explore the relationship between pain sensitisation and pain severity in osteoarthritis patients, and the influence of pain sensitisation on outcomes after joint replacement.

